# Validating plasma biomarkers for distinguishing neurodegenerative and psychiatric disorders

**DOI:** 10.1002/alz.71369

**Published:** 2026-04-21

**Authors:** Marina Boccardi, Leonardo Sacco, Daniele Altomare

**Affiliations:** ^1^ Center of Competence on Ageing, Department of Business Economics, Health and Social Care University of Applied Sciences and Arts of Southern Switzerland (SUPSI) Manno Switzerland; ^2^ Department of Neurology Neuropsychology and Speech Therapy Unit, Neurocenter of Southern Switzerland Ente Ospedaliero Cantonale Lugano Switzerland; ^3^ Faculty of Biomedical Sciences Università della Svizzera Italiana Lugano Switzerland

1

The timely paper by Eratne et al.^1^ confirmed expectations and preliminary evidence that plasma neurofilament light chain accurately distinguishes neurodegenerative disorders from primary psychiatric disorders, and that plasma phosphorylated tau 217 distinguishes Alzheimer's disease (AD) from behavioral variant frontotemporal dementia and primary psychiatric disorders. Being able to detect neurodegenerative disorders in populations with misdiagnosed or unsolved cases, referrable to quaternary clinics like Eratne's, is relevant. Indeed, psychiatry clinics may under‐investigate neurodegenerative conditions due to high prevalence of psychiatric disorders and higher sensitivity of assessments to psychiatric and behavioral, rather than cognitive, symptoms. Moreover, young onset neurodegenerative disorders or non‐primarily cognitive presentations, besides mimicking, may also *add* to existing primary psychiatric disorders, and go unnoticed. The rationale for using plasma biomarkers to help in such contexts is clear.

In this promising perspective, further reinforced by Eratne et al.’s contribution, we take the chance to discuss some methodological challenges well exemplified by this study, that may hamper efficient development. Formal methods for the systematic validation of diagnostic biomarkers^2^ demonstrated useful (Figure 1): from complete wastes of effort^3^ before an adaptation to AD and related neurodegenerative disorders was available,^4^ validation efficiency quadrupled when such adaptation was introduced,^5^, ^6^ and was maximized when a further update^7^ was adopted by already methodologically trained researchers^8^, ^9^ (Figure 1).

**FIGURE 1:**
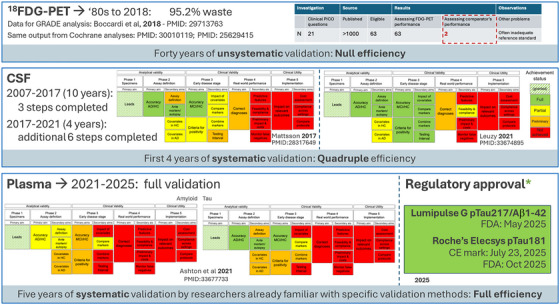
Efficiency of AD biomarker validation increased with availability and compliance with specific systematic methodology. AD, Alzheimer's disease; CE, Conformité Européenne, CSF, cerebrospinal fluid; FDA, US Food and Drug Administration; FDG, fluorodeoxyglucose; GRADE, Grading of Recommendations Assessment, Development and Evaluation; HC, healthy control; MCI, mild cognitive impairment; PET, positron emission tomography; PICO, Population, Intervention/Index test, Comparison, Outcome; ptau, phosphorylated tau

Before claims of clinical transferability, readers familiar with this methodology would easily notice a weakly *representative* population.^9^, ^10^ Eratne et al.’s study involved real‐world patients, hinting at a Phase 4 study (see Boccardi et al.^4^, ^7^ for methodological details). But, consisting of unsolved cases from heterogeneous clinics, their representativeness is limited for any one of them. This leads to the question whether the implied (but undefined) *context of use* consisted of using plasma biomarkers in quaternary clinics to disambiguate over previous specialist assessments, or rather of proposing their incorporation in specialist clinics’ assessments for more exhaustive differential diagnoses, like in the above‐mentioned example of psychiatry clinics. Defining the context of use is key, as it affects the study design of validation steps. The *study design* used by Eratne et al., focusing on a few diagnostic categories selected a priori, enables us to support the rationale for proceeding and validate plasma biomarkers for clinical contexts. However, it does not allow us to *quantify the biomarkers’ informative value* in such contexts: computing accuracy and predictive values requires us to follow up *unselected patients* and keep track of inconsistencies between the target test results with *reference‐standard diagnoses*. These may be primarily clinical in Phase 4 studies; however, confirmation by *consolidated biomarkers* is important, if we still have to quantify the informative value of new biomarkers in a new population. We recognize that real‐world datasets do not always allow us to comply with such complex sets of requirements. However, limitations should be discussed as research priorities and solved before inferring clinical transferability.

We also recognize that applying proper validation methodology^2^, ^4^, ^7^ to new contexts of use requires us to master it well, and sometimes to fold it into unpredicted needs, with solutions that may differ among research groups. At the current state of development, and based on the above considerations, we see Eratne et al.’s purpose to support clinical use of plasma biomarkers in clinics other than memory clinics as requiring a hybrid Phase 3/4 validation study. This should examine *unselected* patients in a specific real‐world context (e.g., psychiatry clinic) as in Phase 4, but without using test results to support clinical diagnosis, and considering consolidated biomarker‐supported diagnoses as reference standard, as required in Phase 3. As it is, Eratne et al.’s study provides explorative evidence encouraging efforts to validate plasma biomarkers for use outside memory clinics, but still lacks definitive evidence to implement them in these contexts.

The complexity of the treated topic raises a further consideration. Many researchers using the validation framework for AD biomarkers took part in two wide international efforts to develop and adopt it (see dedicated issues 52, Neurobiol Aging 2017 and 48, EJNMMI 2021). Adoption by other researchers may require dedicated tools helping them to understand, apply, and master such methodology sufficiently well to devise consistent adaptions to new contexts. To our knowledge, such efforts are scanty to date.

Overall, we greatly appreciated Eratne et al.’s study and the opportunity it gave to discuss old and new hurdles to the clinical validation of diagnostic biomarkers. We hope these considerations provide helpful advice to boost collective progress in the field, improving the compliance of validation studies with methodological requirements. We believe that providing tools to facilitate access, adoption, and consistent adaptations of the methodology itself to new contexts is an equally urgent priority.

## 3 CONFLICT OF INTEREST STATEMENT

M.B. received compensation by OM Pharma for a symposium presentation in November 2025. L.S. is principal investigator in multiple clinical trials, including Celia (phase II, anti‐tau), Trontier (phase III, anti‐amyloid), and the EMERGE and EMBARK studies (anti‐amyloid). L.S. has also participated in advisory boards for Roche, Eli Lilly, Biogen, and Eisai. D.A. has no conflicts of interest. Author disclosures are available in the supporting information.

## 4 CONSENT STATEMENT

This paper required no informed consent.

## Supporting information



Supporting Information
